# Annexin A2 antibodies but not inhibitors of the annexin A2 heterotetramer impair productive HIV-1 infection of macrophages *in vitro*

**DOI:** 10.1186/s12985-016-0649-5

**Published:** 2016-11-18

**Authors:** Andrew W. Woodham, Adriana M. Sanna, Julia R. Taylor, Joseph G. Skeate, Diane M. Da Silva, Lodewijk V. Dekker, W. Martin Kast

**Affiliations:** 1 Whitehead Institute for Biomedical Research, Massachusetts Institute of Technology, Cambridge, MA USA; 2 Norris Comprehensive Cancer Center, University of Southern California, Los Angeles, CA USA; 3 Present Address: Division of Oncology and Pathology, Lund University, Lund, Sweden; 4 Department of Molecular Microbiology & Immunology, University of Southern California, 1450 Biggy St., NRT 7507, Los Angeles, CA 90033 USA; 5 Department of Obstetrics & Gynecology, University of Southern California, Los Angeles, CA USA; 6 School of Pharmacy, Centre for Biomolecular Sciences, University of Nottingham, Nottingham, NG7 2RD UK

**Keywords:** Annexin A2, Annexin A2 heterotetramer, HIV-1, Inhibitor, Macrophage, Receptor

## Abstract

During sexual transmission of human immunodeficiency virus (HIV), macrophages are initial targets for HIV infection. Secretory leukocyte protease inhibitor (SLPI) has been shown to protect against HIV infection of macrophages through interactions with annexin A2 (A2), which is found on the macrophage cell surface as a heterotetramer (A2t) consisting of A2 and S100A10. Therefore, we investigated potential protein-protein interactions between A2 and HIV-1 gp120 through a series of co-immunoprecipitation assays and a single molecule pulldown (SiMPull) technique. Additionally, inhibitors of A2t (A2ti) that target the interaction between A2 and S100A10 were tested for their ability to impair productive HIV-1 infection of macrophages. Our data suggest that interactions between HIV-1 gp120 and A2 exist, though this interaction may be indirect. Furthermore, an anti-A2 antibody impaired HIV-1 particle production in macrophages in vitro, whereas A2ti did not indicating that annexin A2 may promote HIV-1 infection of macrophages in its monomeric rather than tetrameric form.

## Introduction

**Fig. 1:**
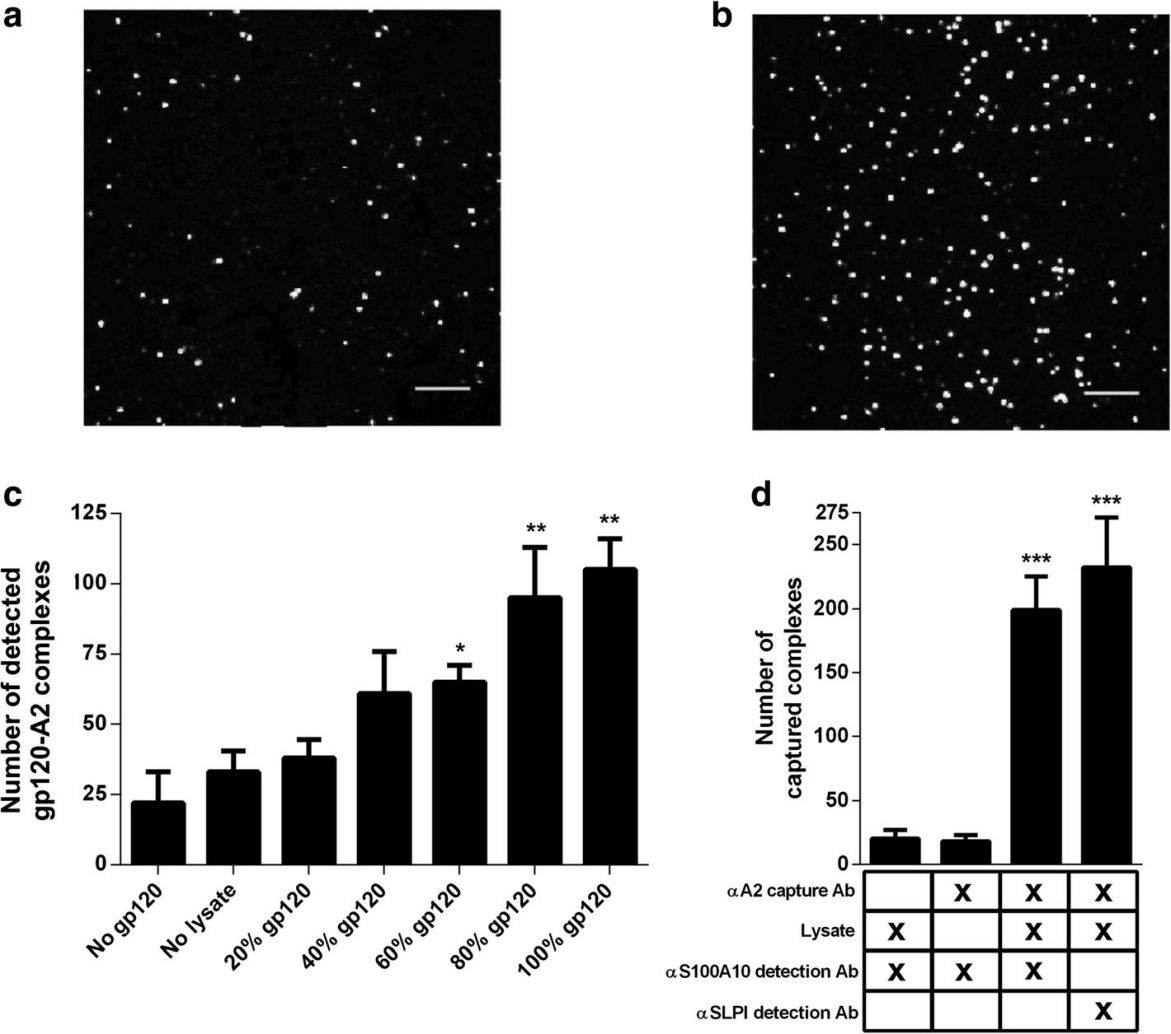
A2 from macrophage lysates is captured on HIV-1 gp120-coated SiMPull slides. Lysis buffer (**a**) or macrophage cell lysates (**b**) were flowed onto SiMPull slides coated with increasing amounts of biotinylated gp120, and the number of captured complexes (**c**) were detected following staining with a rabbit anti-A2 antibody and an anti-rabbit 568-conjugated secondary antibody using TIRF microscopy, where each white dot represents one protein-protein complex (scale bar = 5 μm). Controls included no gp120 and no lysate. Data are presented as the means ± SD of five fields of view of a representative example of an experiment performed three times. *p < 0.05 **p < 0.01 as determined by a one-way ANOVA followed by a Kruskal-Wallis multiple comparisons test against the no gp120 control group. **d** In a separate experiment, lysates were flowed onto SiMPull slides coated with an anti-A2 antibody, and captured complexes were detected with mouse anti-S100A10 or anti-SLPI primary antibodies and an anti-mouse 568-conjugated secondary antibody. ***p < 0.001 as determined by an unpaired two-tailed Student’s *T*-test against the no capture control group

During sexual transmission of human immunodeficiency virus (HIV), macrophages of the cervical, anal, and foreskin epithelium are among the first immune cells to encounter the virus, which makes them initial targets for HIV infection [1, 2]. It is well established that secretory leukocyte protease inhibitor (SLPI), a protein found in high concentrations in mucosal fluids, protects against HIV-1 infection of macrophages independent of its anti-protease activity [3, 4]. Moreover, when the host-cell membrane constituent phospholipid phosphatidylserine (PS) is incorporated into the viral envelope during the budding process, it acts as a cofactor for HIV-1 infection of macrophages [5]. The ability of host-derived PS to influence HIV-1 infection led to the prediction that an unknown factor on target-cell membranes facilitated viral binding and/or fusion through PS. It was later revealed that SLPI directly interacted with annexin A2 (A2), a PS-binding moiety, and that SLPI could disrupt the interaction between A2 and PS on the HIV-1 envelope to prevent infection in vitro [6] (also see Fig. 1d). Additionally, antibodies against A2 or RNA silencing of A2 significantly inhibited HIV-1 infection similar to that of SLPI. It was also shown that A2 is involved in HIV-1 replication in monocyte-derived macrophages (MDMs) [7], and that HIV-1 produced from MDMs that had been treated with A2 siRNA exhibited decreased infectivity [8].

Generally, HIV-1 infects macrophages through the canonical CD4 receptor CCR5 coreceptor pathway [2, 9], though numerous cofactors can affect the efficiency of this process and the rate of infection [5, 6]. Entry inhibitors, such as the CCR5 antagonist maraviroc [10], often lead to the emergence of resistant HIV-1 strains that can use alternative pathways [9]. Moreover, alternative pathways of HIV-1 infection are likely to differ in macrophages and CD4^+^ T cells as they express different membrane components such as PS and A2, which are found on the macrophage cell membrane but not on viable T cells [4, 7]. A2 can be found on the cell surface as a heterotetramer (A2t) consisting of two A2 monomers and an S100A10 dimer [11], which are co-expressed by macrophages [7]. Additionally, data from the HIV-1 Human Interaction Database from the National Center for Biotechnology Information (NCBI) suggests that there may be interactions between HIV-1 gp120 and host A2 [12], though direct evidence is lacking. Recently, our collaborators developed triazole-based small molecule inhibitors of A2t (A2ti) that specifically disrupt the interaction between A2 and S100A10 [13], and we showed that these small molecules block infection of the A2t-utilizing human papillomavirus type 16 (HPV16) [14], but have yet to be explored in the context of HIV. While A2 has already been implicated in HIV-1 infection of macrophages [6, 15], it is not understood if A2t acts as a cofactor for infection. Therefore, the goals of the current study were to investigate potential protein-protein interactions between A2 and the HIV-1 envelope protein gp120, and the ability of A2ti to inhibit HIV-1 infection of macrophages in vitro.

## Methods

HEK293T cells maintained in in DMEM with L-glutamine (Lonza, Walkersville, MD, USA), 10% FBS, and 1% sodium pyruvate were transfected at 50–75% confluence with the HIV-1_JR-CSF_ plasmid using the Calcium Phosphate Transfection Kit (Invitrogen, Life technologies) according to manufacturer instructions. Supernatants containing HIV-1 (viral inoculum) were harvested after 72 h, filtered through 0.45 μM filters, and titered using the GHOST-indicator cell line as previously described [16]. Monocytes were isolated from total peripheral blood mononuclear cells (PBMC) via the EasySep negative selection Human Monocyte Enrichment kit (STEMCELL technologies) according to manufacturer instructions. Isolated monocytes were then used to derive MDMs via 7-day incubation with 1000 U/mL granulocyte-macrophage colony-stimulating factor (GM-CSF) (Leukine, Sanofi-Aventis, Bridgewater, NJ, USA) as previously described [17], and were confirmed as CD11c^+^CD14^−^CD4^low^CCR5^+^A2^+^S100A10^+^ (data not shown). Recombinant A2 and S100A10 proteins were gifts from Ralf Langen [18], and recombinant purified HIV-1 gp120 biotin was purchased from Immune Technology (New York, NY, USA). A2ti were synthesized as described [13].

## Results

Through a series of enzyme-linked immunosorbent assays (ELISA) and co-immunoprecipitation (co-IP) assays, we investigated whether A2 directly interacts with the primary HIV-1 envelope protein gp120. Following published procedures that we utilized for the evaluation of A2 interactions with HPV16 [18], we found that recombinant gp120 was not able to bind to purified A2 or S100A10 when measured via ELISA, and that gp120 incubated with macrophages was unable to co-IP with A2 (data not shown). However, in addition to traditional co-IP assays, we used a highly-sensitive single molecule pulldown (SiMPull) technique that combines classical pulldown methodology with single molecule fluorescence imaging [19]. We found that when biotinylated gp120 (1 μg/mL) was captured onto SiMPull slides as a bait protein via NeutrAvidin [19], and macrophage lysates (protein concentration 0.5 μg/mL) were then added, A2-gp120 complexes were detected with a rabbit anti-A2 antibody (Santa Cruz Biotechnology, Santa Cruz, CA, USA) followed by an anti-rabbit 568-conjugated secondary antibody (Abcam, Cambridge, MA, USA) using total internal reflection fluorescence (TIRF) microscopy. Max gp120 binding to the slides (100%) was determined as the amount of gp120 added at which no more could be detected directly with an anti-gp120 antibody (Immune Technology). The slides were washed after each step to remove unbound gp120, proteins in the lysate, and detection antibodies. The maximum number of A2-gp120 complexes detected was approximately 50% of those detected for A2-S100A10 or A2-SLPI complexes detected in control experiments with an anti-A2 capture antibody (BD Bioscience) and S100A10 (BD Bioscience) and SLPI (R&D Systems) detection antibodies, but was 3-fold higher than background levels (Fig. 1c and d). These results indicate that A2 from macrophage lysates interacts with gp120 in the absence of other viral proteins, though it is possible that this interaction is indirect and is mediated by other binding partners present in macrophage lysate (Fig. 1).

**Fig. 2:**
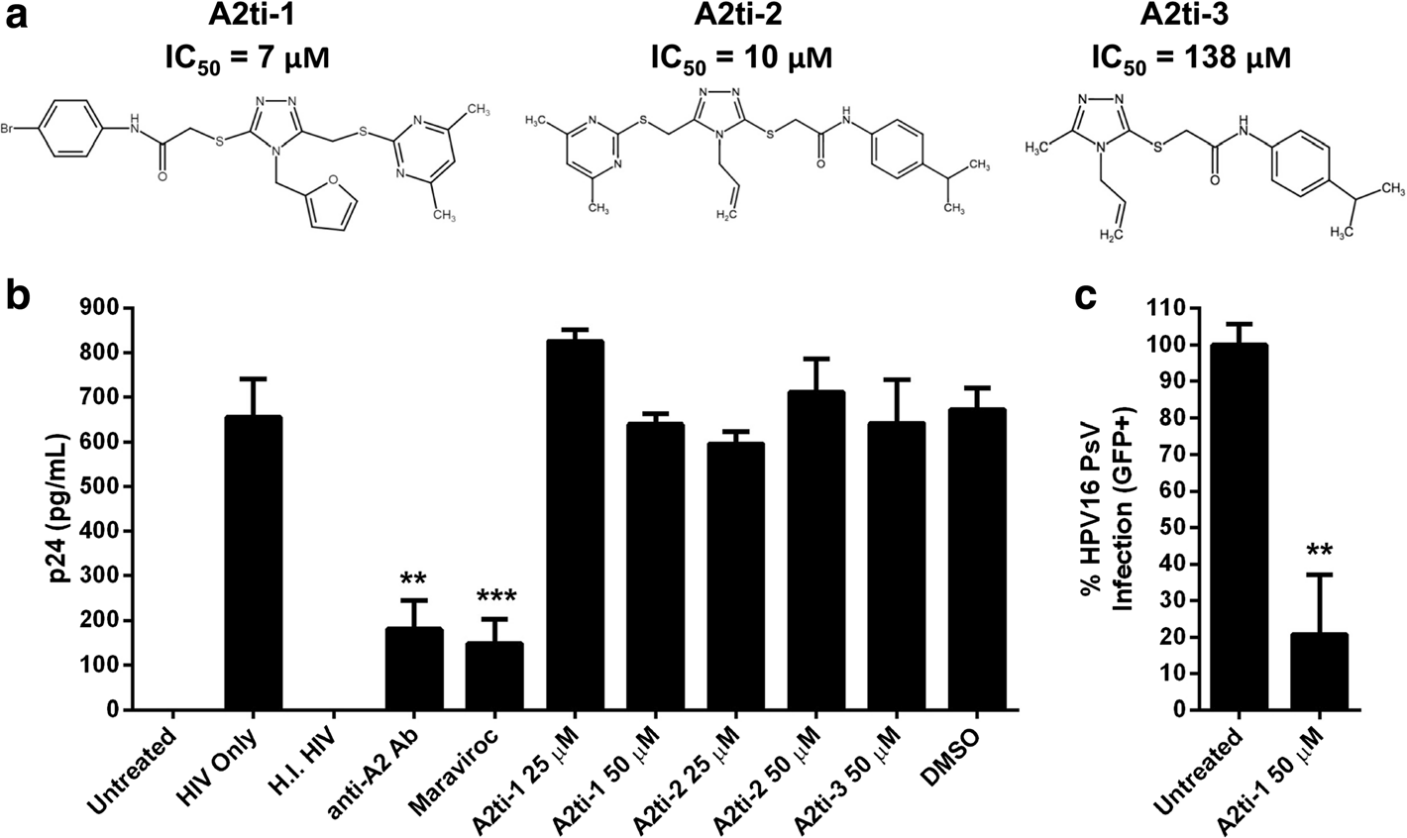
in vitro HIV-1 infection of macrophages following treatment with A2ti, anti-A2 antibody, or maraviroc. **a** Structures and reported IC_50_ values of the three triazole-based A2ti tested [13]. **b** Macrophages were treated with different A2ti (25 or 50 μM), an anti-A2 antibody (25 μg/mL), or maraviroc (25 μg/mL) prior to exposure to HIV-1_JR-CSF_ (MOI = 1). After 24 h, the supernatants were collected and the relative amount of the HIV-1 capsid protein p24 (pg/mL) was measured via ELISA. Controls included uninfected, HIV only, heat-inactivated virus (H.I. HIV), and DMSO at a concentration matched to that of 50 μM A2ti. Data are presented as the means ± SD of three independent experiments. **p < 0.01 and ***p < 0.001 as determined by unpaired two-tailed Student’s *t*-tests compared to the HIV only group. **c** HaCaT cells were left untreated or treated with 50 μM A2ti-1. The following day cells were infected with GFP-plasmid-containing HPV16 pseudovirions (PsV). GFP-positive cells were measured after 48 h by flow cytometry. The mean percentage ± SD of infected cells normalized to the untreated group from a representative experiment performed in triplicate is presented. **p < 0.01 as determined by unpaired two-tailed Student’s *t*-test compared to the untreated group

Next, we validated the effects of an antibody against the N-terminus of A2 on productive HIV-1 infection of macrophages [6]. Macrophages were pre-treated with the anti-A2 antibody (Santa Cruz), or maraviroc (NIH AIDS Research and Reference Reagent Program) for 4 h prior to the addition of HIV-1_JR-CSF_ (MOI = 1), and infection was measured via HIV-1 p24 ELISA (ABL, Kensington, MD, USA) following published procedures [6]. Maraviroc efficiently impaired HIV-1 particle production as demonstrated by a three-fold reduction in p24 detection, which was expected as maraviroc is a CCR5 antagonist known to block HIV-1 infection [10]. The antibody against the N-terminus of A2 (Santa Cruz) also effectively inhibited HIV-1 particle production by macrophages as efficiently as maraviroc at the same concentration (Fig. 2b **)**, indicating that A2 is involved in productive HIV infection of macrophages, which is in agreement with previous results [6]. To assess whether A2 is functional as the A2t heterotetramer or as the A2 monomer in HIV infection, we next determined the effects of three different A2ti that we previously developed, two of which had half maximal inhibitory concentration (IC_50_) values in the low μM range, and one that was chemically analogous, but had a much higher IC_50_ value as a control (Fig. 2a) [13]. We found that viral load as measured by p24 levels did not decrease with any of the A2ti, even at concentrations well above the reported IC_50_ values for the two more potent compounds, compared to that of the untreated group (Fig. 2b). However, in agreement with our previous results and following our published procedures [14], A2ti were able to effectively block A2t-utlizing HPV16 infection of HaCaT epithelial cells, serving as a positive control for A2ti activity (Fig. 2c **,** with data for other A2ti available in [14]).

## Discussion

In summary, an anti-A2 antibody targeting the N-terminus of A2 successfully blocked productive HIV-1 infection of macrophages in vitro, but A2t-targeting drugs did not, indicating that A2 may promote HIV-1 infection of macrophages in its monomeric form. This mechanism is different from HPV infection, which involves the heterotetrameric form of A2 [18]. Additionally, the highly-sensitive SiMPull assay demonstrated that immobilized HIV-1 gp120 captured A2 from macrophage lysates. However, this interaction was not robust enough to detect via standard co-IP techniques, and may be indirectly mediated by unidentified cellular factors. Thus, it is possible that other factors interact with gp120 and A2, and these interactions may be blocked by anti-A2 antibodies. As HIV-1 entry inhibitors function to prevent initial or ongoing spread of infection while the current standards of care largely target viral enzymes to which HIV-1 resistance continues to emerge [20], novel entry inhibitors are of great interest, though future research is required to determine if monomeric A2 is a viable target for such approaches.

## Abbreviations

A2: Annexin A2
A2t: Annexin A2 heterotetramer
A2ti: A2t small molecule inhibitor
Co-IP: Co-immunoprecipitation
ELISA: Enzyme-linked immunosorbent assay
GM-CSF: Granulocyte-macrophage colony-stimulating factor
HIV: Human immunodeficiency virus
HPV16: Human papillomavirus type 16
IC_50_: Half maximal inhibitory concentration
MDM: Monocyte-derived macrophage
PBMC: Peripheral blood mononuclear cell
PS: Phosphatidylserine
SiMPull: Single molecule pulldown
SLPI: Secretory leukocyte protease inhibitor
TIRF: Total internal reflection fluorescence
